# A Novel Just-In-Time-Online-Training for Nasopharyngeal Swab Specimen Collection During the COVID-19 Pandemic

**DOI:** 10.7759/cureus.15944

**Published:** 2021-06-26

**Authors:** Asit Misra, Kristy J Carlson, Christie A Barnes, Samuel K Pate, Benjamin B Stobbe, Jayme R Dowdall

**Affiliations:** 1 Emergency Medicine, University of Nebraska Medical Center, Omaha, USA; 2 Otolaryngology - Head and Neck Surgery, University of Nebraska Medical Center, Omaha, USA; 3 Office of Academic Affairs, Interprofessional Experiential Center for Enduring Learning (iEXCEL), University of Nebraska Medical Center, Omaha, USA

**Keywords:** just-in-time-online-training, covid-19, online training, nasal swab, rural, procedural skills, jit, public health education, public health emergency of international concern, nasopharyngeal swabbing

## Abstract

Introduction: The spread of coronavirus disease 2019 (COVID-19) is controlled by timely detection of infected patients using a nasopharyngeal (NP) swab test, followed by isolation and treatment. One challenge encountered with NP swab collection was to train healthcare providers (HCPs) with different training backgrounds and experience for collecting NP swab specimens across Nebraska, including a sizeable rural area. In-person training for NP swab collection skills was challenging due to social distancing. We developed a Just-In-Time-Online Training (JITOT) and delivered it using Facebook Live (TM) to meet our HCPs' training needs.

Methods: Online training was held on April 21, 2020, and attended by 453 HCPs. A quasi-experimental study based on a survey and a multiple choice questionnaire (MCQ) was conducted to evaluate its effectiveness in improving the participants' knowledge and attitudes.

Results: Group mean knowledge score increased from a pre-test score of 57%-95% in the post-test showing a large effect size (Hedges' *g* = 0.976877). On a five-point Likert scale, the majority (86.21%) of the survey respondents agreed/strongly agreed that this training increased their overall comfort for nasal swab specimen collection as compared to their pre-training comfort (37.93%) with this procedure. The majority of respondents (96.55%) in the post-training evaluation agreed/strongly agreed that "the delivery method was appropriate."

Conclusion: A JITOT session is helpful to teach, demonstrate, clarify doubts, and improve the knowledge and comfort of the participants. It can be quickly delivered using a free social media platform for broader outreach during public health emergencies.

## Introduction

Coronavirus disease 2019 (COVID-19) caused by the novel coronavirus (severe acute respiratory syndrome coronavirus 2, SARS-CoV-2) was declared a global pandemic on March 11, 2020 [1]. One of the most effective strategies to control disease spread is the identification, confirmation, and isolation of COVID-19 patients [2]. One method to detect SARS-CoV-2 is by testing a nasopharyngeal (NP) specimen [3-4]. An inadequate NP swab specimen collection may provide an inaccurate result, and a poor technique may result in the patient's discomfort or cause an iatrogenic injury [5]. To improve test accuracy and patient comfort, training of healthcare providers (HCPs) in large numbers in a short time was necessary. The other challenge was to train HCPs with different training backgrounds and experience for collecting NP swab specimens across Nebraska, including a sizeable rural area. This warranted a platform that is readily available and easy to deliver a Just-In-Time-Online-Training (JITOT).

To overcome this challenge, we offered an innovative approach to meet these immediate training needs by quickly developing a JITOT for NP swab specimen collection that was offered online using a popular free social media platform Facebook Live™. The following describes creating a JITOT, developing the delivery method, disseminating the training, assessing the learners for educational outcomes, and evaluating our JITOT.

## Materials and methods

IRB approval

The Institutional Review Board (IRB) of our university determined that this study does not meet the definition of human subject research and, therefore, was exempted from requiring a review by the IRB (IRB #465-20-EX).

Study design

We conducted a survey-based quasi-experimental educational research study to validate the usefulness of this innovative training method (Pre-test --> Intervention --> Post-test). We hypothesized that this online training would improve the participant's mean post-test knowledge scores, comfort, and confidence levels. The training was open to any HCP who may be expected to collect an NP swab specimen during this public health emergency. An information flyer about the goal, training objectives, date, timing with the pre-test link, and the link to the live session was shared over social media, our websites, and various healthcare agencies in Nebraska.

JITOT development process

We used the basic principles of Just-In-Time training theory to develop this training that includes delivering the right information at the right time using the correct method [6].

Educational Goal: The overarching goal of this training was to improve the safety and accuracy of the procedure.

Learning Objectives: After attending the training, the attendees should be able to:

1. Define the correct head position for nasal swab insertion.

2. Differentiate between proper vs. improper trajectory for swab insertion.

3. Identify the correct depth of swab insertion to obtain the sample safely.

4. Define the swab durability testing protocol.

Educational strategies

Experts from the University of Nebraska Medical Center Otolaryngology Department and Interprofessional Experiential Center for Enduring Learning (iEXCEL™) collaborated to create the educational content and developed the assessment and evaluation tools. A training video was also created to demonstrate the correct method of collecting an NP swab specimen. The team brainstormed to choose a widely available, easily accessible, free, and user-friendly platform to offer this training. We chose Facebook Live™ as it met all the above requirements.

Implementation 

The University of Nebraska Medical Center Otolaryngology Department subject matter experts led this live one-hour training, first showing a video followed by a live demonstration of the correct method of obtaining an NP swab specimen. This was followed by a panel discussion over common mistakes and tips to improve the technique. The panel also answered questions that the attendees asked during the session. Examples of learner-generated questions included: 

"What are the absolute contraindications for nasal swab specimen collection?"

"What is the storage temperature for the specimen, and for how long can you store it before sending it to the lab?"

Training evaluation plan

An evaluation was developed based on the principles of the Kirkpatrick program evaluation model to capture the Level 1 Reaction and Level 2 Learning outcomes [7]. We used a five-point Likert scale (Strongly Disagree-1, Disagree-2, Neutral-3, Agree-4, Strongly Agree-5) based pre-and post-test questionnaire to compare the attitudes (comfort and confidence), and used a three-item multiple choice questionnaire (MCQ) developed by our subject matter experts to compare the knowledge scores for the participants. The pre-and post-test questions were the same for the knowledge and attitude part with additional post-training program evaluation questions added to the post-test (based on the Phillips Return on Investment Methodology for training program evaluation) [8]. 

## Results

**Figure 1:**
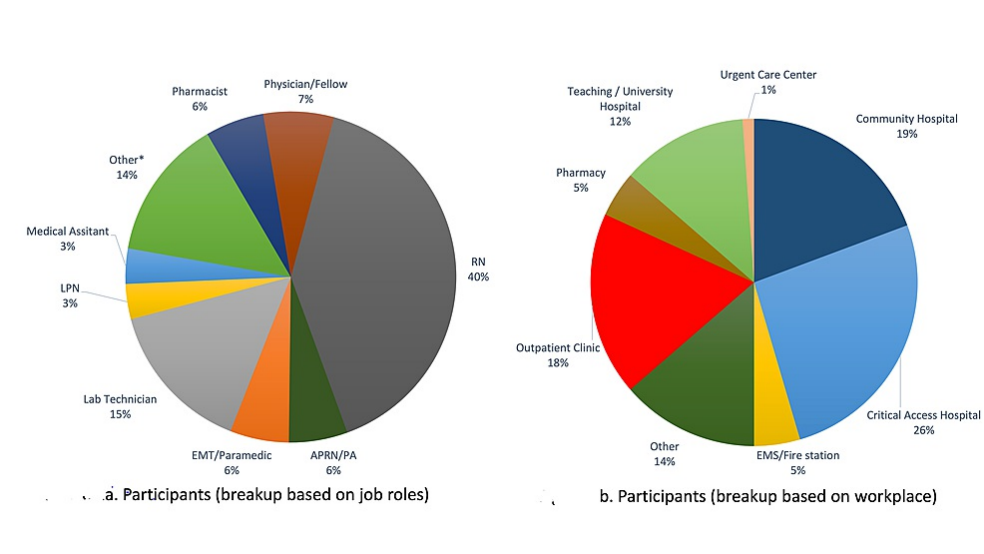
Participant classification based on type of job roles (a) and workplace (b).

**Figure 2:**
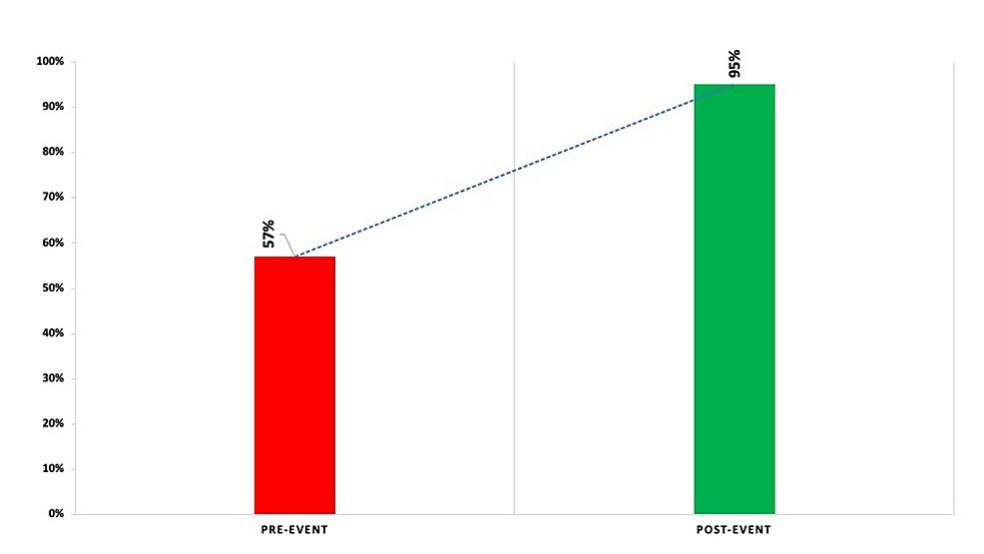
Comparison of total knowledge score Pre- vs. Post-Event (in %).

A total of 87 (19.20%) participants from various healthcare training backgrounds and organizations (Figure 1) completed a voluntary anonymous pre-test, and 29 (6.40%) participants completed a post-test from a total of 453 participants who attended the live session. Analysis of group mean scores (Figure 2) indicated an increase in knowledge score from 57% (pre-test) to 95% (post-test), showing a large effect size (Hedges' *g* = 0.976877).

Post-test results (Table 1) revealed that the majority (86.21%) of the respondents who participated, agreed/strongly agreed that this training increased their overall comfort for nasal swab specimen collection. They also agreed/strongly agreed that this training increased their comfort level for swab durability testing (86.21%), and teaching or demonstrating nasal swab collection techniques to others (89.65%). 

**Table 1:** Pre- vs. Post-training comparison of the participants' comfort and confidence levels. Based on a Likert Scale from 1 = Strongly Disagree to 5 = Strongly Agree.

Attitude and comfort questions (Pre vs. Post)		Pre/Post		Group mean		Standard deviation		Agree/Strongly Agree		(n)
Comfort and demonstration and teaching swab specimen collection	Pre		2.91		1.21		35.64%		87	
	Post		4.21		0.86		89.65%		29	
Ability to differentiate between proper vs. improper trajectory for swab insertion	Pre		2.99		1.12		36.79%		87	
	Post		4.17		0.85		89.65%		29	
Knowledge of correct depth of swab insertion to obtain sample	Pre		3.01		1.08		35.64%		87	
	Post		4.24		0.83		93.10%		29	
Awareness of amount of swab pressure needed to collect specimen	Pre		2.85		1.08		31.39%		86	
	Post		4.31		0.81		96.55%		29	
Comfort in obtaining a nasopharyngeal swab specimen	Pre		2.97		1.13		37.93%		87	
	Post		4.14		0.95		86.21%		29	
Comfort with durability testing of the nasal swab	Pre		2.91		1.21		32.19%		87	
	Post		4.17		0.89		86.21%		29	

**Figure 3:**
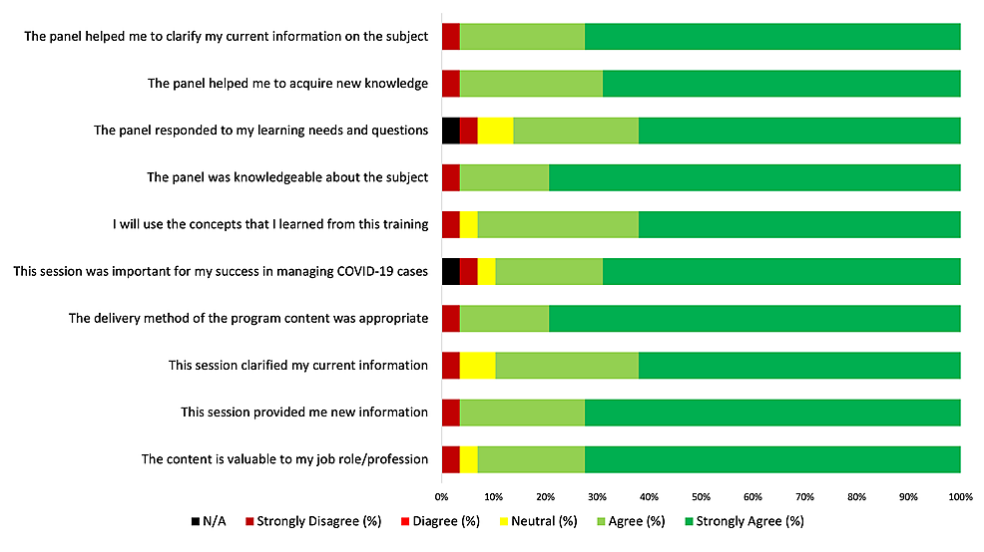
Training evaluation survey results (based on a 5-Point Likert Scale).

Also, in our training evaluation survey (Figure 3) (n=29), 96.55% of respondents agreed/strongly agreed that "the training delivery method was appropriate" and "the panel was knowledgeable about the subject." Further, they agreed/strongly agreed that the training "provided content that is valuable for their job roles" (96.10%); "provided new information that is valuable for them in the current context" (96.55%); "was important for their success in managing COVID-19 cases" (89.66%); “will use the concepts that they learned from this training” (93.10%); and "the panel of experts helped them acquire new knowledge" (96.56%); "panel helped them to clarify their current knowledge on the topic" (96.55%).

## Discussion

An incorrect technique used to collect an NP swab specimen can increase patient discomfort, iatrogenic injury, and/or result in an inadequate sample that may produce false-negative or inconclusive test results [5]. During the COVID-19 pandemic, HCPs in rural Nebraska needed to refresh their procedural knowledge of NP swab specimen collection. A JITOT session via Facebook Live™ offered a real-time demonstration of the correct procedural technique by experts, followed by a panel discussion on tips and challenges with a Q&A session. Four hundred fifty-three people attended this JITOT conducted on April 21, 2020, and the total views of the session (LINK: https://www.facebook.com/UNMC.iEXCEL1/videos/2715108175283603/) were 4,505 as of September 9, 2020. The usefulness of such JITOT is supported by improved post-training knowledge scores, confidence, and attitude levels of participants. This training format was positively perceived by the participants, as indicated in the post-training evaluation responses.

Previous studies have reported successful training outcomes using Just-In-Time training for skill refresh to perform a procedure resulting in improved procedural confidence, performance, patient satisfaction, and clinical outcomes [6, 9-12]. A recent study suggests the use of Just-In-Time training improved HCPs’ competencies (knowledge) to respond during the COVID-19 pandemic [13]. Likewise, we also witnessed improved knowledge scores and confidence to collect NP swab specimens following the training session. This may transform into the correct application of knowledge and procedural skills by the participants while performing an NP swab specimen collection.

Using a free social media platform to deliver this JITOT session has several benefits. Key HCPs and public health officials were identified throughout the State, who then invited others (via personal and organizational Facebook pages) resulting in snowball recruitment. Due to the immediate need to develop the NP swab skill, this advertising technique was practical to quickly disseminate the training to a large number of HCPs. Further, online delivery was ideal for extending training to the rural HCPs who would not otherwise have access to in-person training. Finally, specialty physicians were available in real-time to answer questions regarding technique and patient safety.

We used the basic principles of Just-in-Time training to develop this novel delivery method that is JITOT [6]. Educators and curriculum developers may find some of the following tips helpful in setting up JITOT:

1) Develop and deliver training in a limited time 

2) Keep the development and delivery cost low

3) Choose the right platform for broader reach 

4) Use the resources that are already available (collaboration)

5) Select the correct level of training evaluation to measure the success.

Our training evaluation indicated that more than 90% of the respondents (n=29) either agreed or strongly agreed regarding the value of the training content to their job, received new information, clarified their current information, used appropriate delivery method of the training, and will use the concepts they learned at their job. Further, more than 95% of the respondents agreed/strongly agreed that the experts were knowledgeable, helped them acquire new knowledge, and helped them clarify their current knowledge. 

In future pandemics and public health emergencies, the JITOT framework and free online delivery used in this study could be replicated for new skill development or refresher training. We also found some limitations in our study such as registration to access the Facebook Live platform may have limited participation, a small group could view the training using one individual’s log-in. Increases in knowledge and comfort/confidence level were observed; however, paired analysis was not possible to examine the impact on individual learners as anonymous responses were recorded instead a comparison of group mean score was done. Additionally, assessing the engagement of the learners was difficult due to the online delivery mechanism (i.e., no webcam or video disabled). We also witnessed a drop in the number of participants who took the post-event MCQ test and filled out the survey questionnaire. Post-test and training evaluation survey was an optional component of this training and might be a reason for the low responses. This is not uncommon in educational research and quality improvement studies. 

The responses helped us understand that this novel method is helpful in reviewing and improving knowledge, adding comfort, and confidence in performing a procedural skill when traditional methods of training are not available. Future studies using experimental educational research design are needed to compare the effectiveness of our novel JITOT framework vs. traditional training delivery method during an ongoing public emergency/pandemic.

## Conclusions

This novel JITOT method offered only support that was needed at the appropriate time to train a diverse group of rural HCPs in response to a public health emergency, when in-person and the hands-on training was limited due to social distancing in place. Our JITOT helped revise or improve the participants' procedural knowledge and attitudes (comfort and confidence) related to the NP swab specimen collection by watching a video (live demonstration), animation, and listening to the panel discussion. This low-cost JITOT is effective and could be easily replicated to provide training for a broad audience using a free social media platform to serve their communities quickly.
